# Vitamin D Status and Its Association with Multiple Intelligence among Arab Adolescents

**DOI:** 10.3390/ijerph182413036

**Published:** 2021-12-10

**Authors:** Ahmed S. Mohammed Metwally, Sobhy M. Yakout, Malak N. K. Khattak, Ghadah Alkhaldi, Nasser M. Al-Daghri

**Affiliations:** 1 Department of Mathematics, College of Science, King Saud University, Riyadh 11451, Saudi Arabia; dalsayed@ksu.edu.sa; 2 Biochemistry Department, King Saud University, Riyadh 11451, Saudi Arabia; sobhy.yakout@gmail.com (S.M.Y.); malaknawaz@yahoo.com (M.N.K.K.); 3 Department of Community Health Sciences, College of Applied Medical Sciences, King Saud University, Riyadh 11451, Saudi Arabia; ghalkhaldi@ksu.edu.sa

**Keywords:** secondary school, multiple intelligence, vitamin D

## Abstract

Studies investigating the association of vitamin D on intelligence is limited. The present study therefore aims to determine the association of vitamin D status with the different domains of intelligence among Saudi Arabian adolescents. This study used relational survey method among 1864 Saudi adolescent, including 549 boys and 1315 girls (mean age 14.7 ± 1.7 years) recruited using a multistage, stratified cluster randomization of 47 public and private schools in Riyadh in Saudi Arabia. A general questionnaire was used to collect demographic information. Intelligence was assessed using multiple intelligence inventory. Anthropometrics were measured and fasting blood samples collected for assessment of glucose and lipid profile. Vitamin D deficiency (25(OH)D <50 nmol/L) was observed in 84.2% of boys and 93.5% of girls. Girls had higher levels of verbal, kinesthetic, musical, naturalist and existential intelligence than boys, while boys have higher logical intelligence than girls (*p*-values < 0.05). Mixed regression analysis controlled for age, BMI and sex revealed that kinesthetic intelligence was significantly associated with 25(OH)D in boys (β 5.6 (2.8–8.5; *p* < 0.001)) and inversely associated with musical intelligence (β −1.2 (−2.3–0.1; *p* = 0.03)) and positively with naturalist (β 2.3 (0.5–4.2; *p* = 0.01)) in girls. Vitamin D status is associated with several domains of intelligence in adolescents and is sex-specific. Development a specific domain of intelligence may indirectly affect vitamin D status among adolescents, but needs to be proven prospectively.

## 1. Introduction

Intelligence is the capacity to learn new knowledge and skills to solve problems. It reveals the ability to understand environments [1]. Most definitions of intelligence focus on academic success [1,2], but it is acknowledged that other components of intelligence such as problem-solving cannot be probed sufficiently in short-answer tests [3], hence the need for other assessment tools that go beyond conventional measures of acquired skills [3,4,5].

Based on psychometric as well as neuropsychological evidence, Gardner suggested that there are multiple intelligences or cognitive abilities in normal individuals as well as “special populations” such as prodigies, savants, autistic individuals and learning-disabled children (modern multiple intelligence theory) [6,7]. Gardner described nine types of intelligence: (i) linguistic, (ii) logical, (iii) spatial, (iv) interpersonal, (v) naturalistic, (vi) intrapersonal, (vii) kinesthetic, (viii) musical and (ix) existential, all of which interact and work together in complex ways [8]. These multiple intelligences when assessed correctly can aid instructors identify the students’ maximum potential [9,10].

The theory of multiple intelligence suggests that each person has varying capacity in all nine intelligences and everyone has the ability to develop all if given the suitable environment and conditions [10]. The application of this theory is crucial in maximizing human cognitive capability and efficiency [11]. Cultivating each type of intelligence in students elevates academic performance [12,13] and identifies the gifted individuals [14,15,16].

Vitamin D is an essential micronutrient important for calcium homeostasis and bone growth. The extra-skeletal effects of vitamin D has been of great interest in the recent years, with vitamin D status affecting almost every aspect of metabolism from fetal development until adulthood [17,18,19], including major outcomes to diseases and even epidemics [20,21,22,23]. Furthermore, vitamin D has several biological actions related to neurodevelopment and brain function [24]. A strong relationship between vitamin D deficiency, cognitive function and behavior have been documented in animal models and observational studies [25]. Vitamin D receptor (VDR) and enzymes necessary for vitamin D activation are found in the brain which can convert 25(OH)D into 1,25(OH)2D, the active metabolite [26]. This can influence several neurologic processes including cell differentiation, neurotransmitter synthesis, antioxidant effects, intracellular calcium homeostasis, neuronal development, metabolism and cognitive function [26]. Sunlight represents one of the main sources for vitamin D and is also found in some food sources [27]. On a global level, vitamin D deficiency is a major public health problem [28], including Saudi Arabia (SA) where despite abundant sunshine, there is still a high prevalence of vitamin D deficiency (SA) at 73.2% [29]. The (Middle East and North Africa) MENA region on the other hand, has vitamin D deficiency prevalence ranging from 54% to 90% in adults [30].

Studies investigating the relationship between vitamin D status and cognitive function are mostly observational with mixed results from scant clinical trials [31]. Nevertheless, evidence from the last 16 years concerning people with learning disabilities were recently reviewed and found that vitamin D deficiency prevalence in this population ranges from 49–77%, considerably higher than the general population [32]. Another recent study observed that maternal vitamin D status is positively linked to intelligence quotient (IQ) in children aged 4–6 years [33]. These studies while suggestive, still needs to be taken into context, given the numerous factors that can affect cognition. Nevertheless, there is still a need to further identify the extent of vitamin D status’ influence on brain health, including intelligence. To the best of our knowledge, the association of vitamin D status with the different domains of intelligence has never been investigated. Such studies may offer new insights as to which domains of intelligence can be influenced by micronutrient deficiencies such as vitamin D deficiency. To fill this gap, the purpose of the present study is to examine if there is an association between vitamin D status with the different types of intelligence among Arab adolescents.

## 2. Materials and Methods

### 2.1. Participants

A total of 1864 apparently healthy Saudi adolescent boys (N = 549) and girls (N = 1315) aged 13–17 (mean age 14.7 ± 1.7 years) from different public, private schools within Riyadh, SA were invited to participate in this cross-sectional study from November 2019 until March 2020 before the national lockdown was imposed. The capital Riyadh, from where the sampling frame was taken, is divided into four regions (North, South, East and West). A multistage stratified cluster random technique was then used to recruit the participants wherein schools were chosen randomly in each region, and one class is randomly selected from each grade level, leaving the other classes, and all students from the selected class were encouraged to participate. Consent from parents and assent from students were obtained prior to inclusion.

Sample size calculation was done online (Raosoft.com) given 2% margin of error at 95% confidence interval and 10% response distribution in Riyadh, the minimum recommended sample size should be N = 829. Out of the 2650 students invited, 1864 consented to participate and provide fasting blood samples (70.3% response rate).

**Figure 1:**
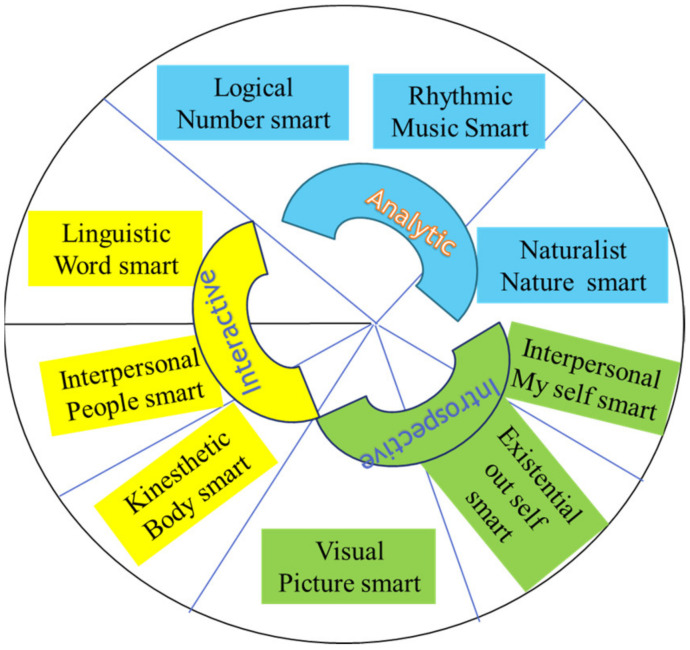
Multiple Intelligence Domains.

Ethical approval was obtained from the Ethics Committee of the College of Science Research Center, King Saud University, Riyadh, SA (No. E-19-239 on 29 October 2019). Two questionnaires were administered to all students: the demographic questionnaire and the McKinsey Multiple Intelligence (MI) Inventory [34,35], to identify the typology of intelligence. The introspective domain consists of existential, intrapersonal and visual intelligences (Figure 1). The second part had 90 questions in Likert scale format (1-2-3-4-5) to assess the nine intelligences proposed by Gardner [6]: verbal (Linguistic), logical, visual, kinesthetic, musical (rhythmic), intrapersonal, interpersonal and naturalist existential. MI can be divided to three domains: the analytical, introspective and interactive domains. These domains help in understanding the fluid relationship of the intelligences and how they work with one another. Figure 1 presents each domain and its sub-branches in details.

### 2.2. Anthropometric and Biochemical Parameters

Anthropometric data and fasting blood extraction were performed in schools at a designated date and facilitated by the school physician (if available) or well-trained research nurses working in collaboration with the Chair for Biomarkers of Chronic Diseases (CBCD) in King Saud University (KSU), Riyadh, SA. Anthropometrics included weight (kg), height (cm), waist (cm), hips (cm) and blood pressure (mmHg). Fasting blood samples (5 cc) was collected and stored using non-heparinized tubes and transported immediately to CBCD in KSU, Riyadh, KSA for storage at −20°C. Fasting blood glucose and lipid profile (triglycerides, total and HDL-cholesterol) were assessed using Konelab (Vintaa, Finland). LDL-cholesterol was calculated using the Friedewald formula. Serum 25-Hydroxyvitamin (OH) D was measured using COBAS e-411 automated analyzer (Roche Diagnostics, Indianapolis, IN, USA) in a DEQAS-certified laboratory (CBCD) [29]. For serum 25-hydroxyvitamin D assay, the inter- and intra-assay coefficients of variation (CV) were 8.0% and 5.6%, respectively, with a lower detection limit (LOD) of <4 ng/mL) [29]. Vitamin D deficiency was defined as 25(OH)D <50 nmol/L [19,20].

### 2.3. Data Analysis

Data were analyzed using SPSS (version 24 Chicago, IL, USA). Continuous data were presented as mean ± standard deviation (SD) for normal variables and non-normal variables were presented in median (1st and 3rd) percentiles. Categorical data were presented as frequencies. All continuous variables were checked for normality using Kolmogorov–Smirnov test. Independent T-test was used to compare mean differences. Correlations between variables were done using Pearson’s correlation analysis. Mixed-Effect regression analysis was performed using 25(OH)D as dependent variable and MI as independent variables, controlled for age and BMI and stratified according to sex. A *p*-value < 0.05 was considered statistically significant.

## 3. Results

The demographic and clinical characteristics as well as MI scores of boys and girls are shown in Table 1. Vitamin D deficiency was noted in 84.2% of the boys and 93.5% of the girls (not shown in table). Boys were significantly older, had higher BMI, WHR, glucose, HDL, triglycerides and 25(OH)D than girls (*p*-values < 0.05) while girls had higher total cholesterol than boys (*p* = 0.04). In terms of MI, girls have significantly higher verbal (*p* = 0.02), kinesthetic (*p* = 0.047), musical (*p* < 0.001) naturalist (*p* = 0.019) and existential intelligences (*p* = 0.004) than boys. However, boys have only significantly higher logical intelligences (*p* = 0.03). No further significant differences were found.

**Figure 2:**
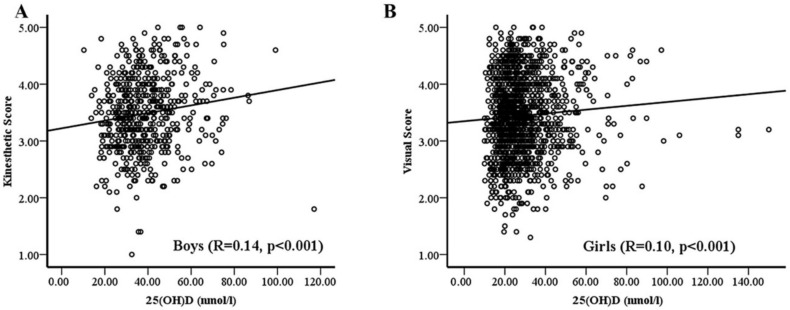
Unadjusted association of 25(OH)D with Kinesthetic Intelligence in Boys (**A**) and Visual Intelligence in Girls (**B**).

Table 2 shows the bivariate associations of 25(OH)D with MI in boys and girls. In all subjects, 25(OH)D showed a significant positive correlation with logical (*p* < 0.01), kinesthetic and intrapersonal intelligences as well as a significant inverse correlation with musical intelligences (*p*-values <0.05). Stratification according to sex revealed that in boys, 25(OH)D was positively associated with logical (*p* < 0.05) and kinesthetic (*p* < 0.01) intelligences while in girls, 25(OH)D was significantly associated with verbal, logical, visual, kinesthetic, naturalist and introspective (*p*-values <0.05). In terms of domain, girls have significantly higher (*p* < 0.05) introspective domain than boys (Table 2). Figure 2 shows the bivariate association between MI and 25(OH)D which showed the most significant correlation in boys (2A) and girls (2B).

Mixed regression analysis using 25(OH)D as dependent variable with different MI as independent variables controlled for age and BMI, stratified for sex are shown in Table 3. Overall, verbal (β-2.2 (−3.9–−0.5; *p* = 0.01)) and musical intelligences (β-1.3 (−2.2–−0.4; *p* = 0.004)) showed a significant inverse association with 25(OH)D. Stratified according to sex, only kinesthetic intelligence was significantly associated with 25(OH)D in boys (β 5.6 (2.8–8.5; *p* < 0.001)) while in girls, 25(OH)D was inversely associated with musical intelligence (β −1.2 (−2.3–0.1; *p* = 0.03)) and positively with naturalist (β 2.3 (0.5–4.2; *p* = 0.01)). The rest of the associations were not significant.

## 4. Discussion

The present study found that differences exist in MI of boys and girls, and that certain types of intelligence is associated with vitamin D status independent from age and BMI. Girls have higher levels of verbal, kinesthetic, musical, naturalist and existential intelligence than boys, who in turn, have higher logical intelligence. An extensive body of literature have documented sexual dimorphism in the estimation of MI, which the present study confirms [36,37,38,39]. However, other studies indicate no apparent sex-differences either on subscale level or total MI score. Our results are similar to a study done in Spain [40], in that Spanish boys had more logical-mathematical and interpersonal intelligence than girls, which was in contrast to a study done among Turkish children [41]. Whether the differences in MI of boys and girls have more to do with culture and education system variations across ethnic groups rather than biological needs further clarification.

The present study also showed that majority of students living in Riyadh are vitamin D deficient, with approximately 84.2% of the boys and 93.5% of the girls having 25(OH)D levels below 50 nmol/L. This is consistent with recent local studies pointing to high prevalence of vitamin D deficiency among adolescents in Saudi Arabia was around 96% [29,42]. Even among newborns, a study conducted in 2017 showed that 88% of newborns had levels of 25(OH) D <50 nmol/L [43]. Another study from our group in 331 Saudi children aged 6–17 years (153 boys and 178 girls) demonstrated that all subjects were vitamin D deficient (serum 25-(OH) vitamin D) with the majority being moderately deficient (71.6%) [44]. Increased prevalence among school aged children and adolescents has been reported, reflecting modern-day lifestyle changes, lack of knowledge about vitamin D sources (sun exposure and food) mostly among girls. The other explanation could be the fat distribution, with females having more fat percentage, decreasing vitamin D bioavailability [45].

Using mixed regression analysis, the present study showed a significant association between vitamin D status and kinesthetic intelligence in boys and naturalist intelligence in girls. It is established that males are more physically active than females and this difference is more pronounced in adolescents [46], affecting kinesthetic intelligence. People with high kinesthetic intelligence more likely to perform sports activities which may require large spaces such as outdoor venues, thus acquiring vitamin D from its natural sources. The same concept may apply to girls, since the development of naturalist and visual intelligence require knowledge from different sceneries and natural environments, consequently influencing endogenous vitamin D levels from such outdoor exposures. It is most likely therefore that the significant associations to 25(OH)D elicited are an indirect consequence of enhancing these domains of intelligences and not vice versa. Potential mediators and predictors of these associations may include, but not limited to, physical activity, diet and socioeconomic status, all of which were not captured in the model. The last potential factor is particularly important as childhood socioeconomic status are associated with both vitamin D status and degree of psychometric intelligence [47,48]. Nevertheless, vitamin D is considered as an essential neuro-steroid with several brain actions [49]. Circulating vitamin D crosses brain barrier to be converted into active form of vitamin D (1,25(OH)2D) [50]. One study observed the influence of vitamin D on neurotransmitter changes and concluded that vitamin D deficiency alters brain changes [51]. In a review done by Freedman et al., there is a relation between micronutrient supplementation during pregnancy and childhood mental illness [52].

The results of the present study should be interpreted with caution taking into full consideration its limitations. First is the cross-sectional design which prevents causality among correlations elicited. Several factors affecting vitamin D status such as season, physical activity, socioeconomic status and dietary intake were also not included in the analysis and this can affect the strength of associations aside from the well-established covariates such as age and BMI. While some associations elicited between vitamin D status and MI were significant, the association itself is weak and as such the results are at most, suggestive. Lastly, the present results may only apply to adolescents, and associations of vitamin D status to MI maybe totally different in other populations in different life stages.

## 5. Conclusions

Among Arab adolescents, several types of MI are associated by vitamin D status in boys and girls, independent of age and BMI. Girls had higher levels of verbal, kinesthetic, musical, naturalist and existential intelligences than boys and boys have higher logical intelligence than girls. Vitamin D status is associated with kinesthetic intelligence in boys and naturalist intelligence in girls independent of age and BMI. These associations with 25(OH)D may be an indirect consequence of developing specific domains of intelligence that require outdoor environmental exposures. Prospective studies may determine whether vitamin D correction among adolescents may improve development of several types of MI.

## Figures and Tables

**Table 1:** Differences in Clinical Characteristics and MI Scores of Boys and Girls.

Parameters	All	Boys	Girls	*p*-Value
N	1864	549	1315	
Age (years)	14.7 ± 1.7	15.1 ± 1.6	14.6 ± 1.7	<0.001
BMI (kg/m^2^)	23.1 ± 6.7	23.9 ± 6.7	22.8 ± 6.7	0.003
WHR	0.81 ± 0.1	0.88 ± 0.1	0.79 ± 0.1	<0.001
Systolic BP (mmHg)	118.5 ± 16.2	119.6 ± 14.6	118.1 ± 16.8	0.08
Diastolic BP (mmHg)	72.4 ± 12.0	67.3 ± 10.5	74.6 ± 11.9	<0.001
Glucose (mmol/L)	5.2 ± 0.6	5.3 ± 0.6	5.1 ± 0.5	<0.001
T-Cholesterol (mmol/L)	4.4 ± 0.7	4.3 ± 0.8	4.4 ± 0.7	0.04
HDL-Cholesterol (mmol/L)	0.98 ± 0.2	0.99 ± 0.2	0.97 ± 0.2	0.03
Triglycerides (mmol/L)	1.1 ± 0.5	1.2 ± 0.6	1.1 ± 0.4	<0.001
25(OH) D (nmol/L)	31.8 ± 14.4	38.7 ± 13.5	28.9 ± 13.8	<0.001
Verbal (Linguistic)	3.4 ± 0.6	3.3 ± 0.6	3.4 ± 0.7	0.02
Logical	3.4 ± 0.7	3.5 ± 0.7	3.4 ± 0.7	0.03
Visual	3.4 ± 0.7	3.4 ± 0.6	3.5 ± 0.7	0.08
Kinesthetic	3.5 ± 0.7	3.5 ± 0.6	3.6 ± 0.7	0.047
Musical (Rhythmic)	3.2 ± 0.8	3.0 ± 0.8	3.3 ± 0.8	<0.001
Intrapersonal	3.7 ± 0.6	3.7 ± 0.6	3.7 ± 0.6	0.87
Interpersonal	3.5 ± 0.6	3.5 ± 0.6	3.5 ± 0.6	0.12
Naturalist	3.4 ± 0.6	3.3 ± 0.6	3.4 ± 0.6	0.02
Existential	3.5 ± 0.7	3.5 ± 0.7	3.6 ± 0.7	0.004
Analytic	3.3 ± 0.6	3.3 ± 0.5	3.4 ± 0.6	0.002
Interactive	3.5 ± 0.6	3.4 ± 0.6	3.5 ± 0.6	0.26
Introspective	3.6 ± 0.6	3.5 ± 0.6	3.6 ± 0.5	0.07

**Note**: Data presented as coefficient (R), significant at *p* < 0.05.

**Table 2:** Bivariate Associations of 25(OH)D with MI in Boys and Girls.

Parameters	All	Boys	Girls
N	1864 (549/1315)	549	1315
Verbal	0.00	0.05	0.02 *
Logical	0.06 **	0.09 *	0.06 *
Visual	0.04	0.04	0.10 **
Kinesthetic	0.05 *	0.14 **	0.07 **
Musical	−0.05 *	0.03	−0.01
Intrapersonal	0.05 *	0.08	0.04
Interpersonal	0.03	0.02	0.03
Naturalist	0.04	0.01	0.08 **
Existential	0.01	0.06	0.03
Analytic	0.02	0.06	0.05
Interactive	0.03	0.08	0.05
Introspective	0.04	0.07	0.06 *

**Note**: Data presented as coefficient (R), * denotes significance at 0.05 level; ** denotes significance at 0.01 level.

**Table 3:** Mixed-Effect Regression Analysis for Vitamin D Status and MI.

Parameters	All		Boys		Girls	
	β (95% CI)	*p*-Value	β (95% CI)	*p*-Value	β (95% CI)	*p*-Value
Verbal	−2.20 (−3.9–−0.5)	0.01	1.58 (−4.7–1.5)	0.32	−1.03 (−3.0–0.9)	0.30
Logical	1.77 (0.2–3.3)	0.03	2.27 (−0.6–5.2)	0.13	−0.07 (−1.8–1.7)	0.94
Visual	0.72 (−0.9–2.3)	0.38	−2.51 (−5.7–0.7)	0.12	1.56 (−0.2–3.3)	0.08
Kinesthetic	1.44 (−0.1–3.0)	0.07	5.64 (2.8–8.5)	<0.001	0.48 (−1.2–2.2)	0.58
Musical	−1.33 (−2.2–−0.4)	0.004	0.99 (−0.5–2.5)	0.2	−1.19 (−2.3–0.1)	0.03
Intrapersonal	1.05 (−0.4–2.6)	0.17	0.66 (−2.2–3.5)	0.65	0.92 (−0.8–2.6)	0.28
Interpersonal	0.24 (−1.3–1.8)	0.77	−2.26 (−5.0–0.5)	0.11	−0.48 (−2.3–1.3)	0.60
Naturalist	0.53 (−1.1–2.2)	0.52	−1.45 (−4.3–1.4)	0.32	2.31 (0.5–4.2)	0.01
Existential Emotional	−1.48 (−3.0–0.1)	0.06	0.06 (−2.6–2.8)	0.96	−1.45 (−3.2–0.3)	0.10

**Note**: Data presented as coefficient β (95% CI); *p*-values adjusted for age and BMI; significant at <0.05.

## Data Availability

The data concerning this study is available from the corresponding author on reasonable request.

## References

[1] Ackerman P.L., Beier M.E., Boyle M.O. (2005). Working memory and intelligence: The same or different constructs?. Psychol. Bull..

[2] Dehn N., Schank R. (1982). Handbook of Human Intelligence.

[3] Cattell R.B., Cattell A.K.S. (1960). Measuring Intelligence with the Culture Fair Tests.

[4] Sternberg R.J. (2000). The Theory of Successful Intelligence. Gifted Educ. Int..

[5] Campione J.C., Brown A.L. (1978). Toward a theory of intelligence: Contributions from research with retarded children. Intelligence.

[6] Gardner H. (1979). Development psychology after Piaget: An approach in terms of symbolization. Hum. Dev..

[7] Hatch T.C., Gardner H. (1986). From testing intelligence to assessing competences: A pluralistic view of intellect. Roeper Rev..

[8] Colom R., Karama S., Jung R.E., Haier R.J. (2010). Human intelligence and brain networks. Dialogues Clin. Neurosci..

[9] Pellegrini A.D. (1988). Psychological Bases for Early Education.

[10] Howe M.J. (1990). Encouraging the Development of Exceptional Skills and Talents.

[11] Schutte N.S., Malouff J.M., Hall L.E., Haggerty D.J., Cooper J.T., Golden C.J., Dornheim L. (1998). Development and validation of a measure of emotional intelligence. Personal. Individ. Differ..

[12] Hille K., Gust K., Bitz U., Kammer T. (2011). Associations between music education, intelligence, and spelling ability in elementary school. Adv. Cogn. Psychol..

[13] Miendlarzewska E.A., Trost W.J. (2014). How musical training affects cognitive development: Rhythm, reward and other modulating variables. Front. Neurosci..

[14] Nuallaong W., Nuallaong T., Preechadirek N. (2015). Academic achievement from using the learning medium via a tablet device based on multiple intelligences in grade 1 elementary student. J. Med. Assoc. Thai.

[15] Sheahan L., While A., Bloomfield J. (2015). An exploratory trial exploring the use of a multiple intelligences teaching approach (MITA) for teaching clinical skills to first year undergraduate nursing students. Nurse Educ. Today.

[16] Hearne D., Stone S. (1995). Multiple intelligences and underachievement: Lessons from individuals with learning disabilities. J. Learn. Disabil..

[17] Al-Musharaf S., Fouda M.A., Turkestani I.Z., Al-Ajlan A., Sabico S., Alnaami A.M., Wani K., Hussain S.D., Alraqebah B., Al-Serehi A. (2018). Vitamin D Deficiency Prevalence and Predictors in Early Pregnancy among Arab Women. Nutrients.

[18] Al-Daghri N.M., Al-Saleh Y., Aljohani N., Alokail M., Al-Attas O., Alnaami A.M., Sabico S., Alsulaimani M., Al-Harbi M., Alfawaz H. (2015). Vitamin D Deficiency and Cardiometabolic Risks: A Juxtaposition of Arab Adolescents and Adults. PLoS ONE.

[19] Al-Daghri N.M., Torretta E., Capitanio D., Fania C., Guerini F.R., Sabico S.B., Clerici M., Gelfi C. (2017). Intermediate and low abundant protein analysis of vitamin D deficient obese and non-obese subjects by MALDI-profiling. Sci. Rep..

[20] Al Saleh Y., Beshyah S.A., Hussein W., Almadani A., Hassoun A., Al Mamari A., Ba-Essa E., Al-Dhafiri E., Hassanein M., Fouda M.A. (2020). Diagnosis and management of vitamin D deficiency in the Gulf Cooperative Council (GCC) countries: An expert consensus summary statement from the GCC vitamin D advisory board. Arch. Osteoporos..

[21] Alguwaihes A.M., Al-Sofiani M.E., Megdad M., Albader S.S., Alsari M.H., Alelayan A., Alzahrani S.H., Sabico S., Al-Daghri N.M., Jammah A.A. (2020). Diabetes and Covid-19 among hospitalized patients in Saudi Arabia: A single-centre retrospective study. Cardiovasc. Diabetol..

[22] Alguwaihes A.M., Sabico S., Hasanato R., Al-Sofiani M.E., Megdad M., Albader S.S., Alsari M.H., Alelayan A., Alyusuf E.Y., Alzahrani S.H. (2021). Severe vitamin D deficiency is not related to SARS-CoV-2 infection but may increase mortality risk in hospitalized adults: A retrospective case-control study in an Arab Gulf country. Aging Clin. Exp. Res..

[23] Sabico S., Enani M.A., Sheshah E., Aljohani N.J., Aldisi D.A., Alotaibi N.H., Alshingetti N., Alomar S.Y., Alnaami A.M., Amer O.E. (2021). Effects of a 2-Week 5000 IU versus 1000 IU Vitamin D3 Supplementation on Recovery of Symptoms in Patients with Mild to Moderate Covid-19: A Randomized Clinical Trial. Nutrients.

[24] Ibi M., Sawada H., Nakanishi M., Kume T., Katsuki H., Kaneko S., Shimohama S., Akaike A. (2001). Protective effects of 1 alpha,25-(OH)(2)D(3) against the neurotoxicity of glutamate and reactive oxygen species in mesencephalic culture. Neuropharmacology.

[25] Etgen T., Sander D., Bickel H., Sander K., Forstl H. (2012). Vitamin D deficiency, cognitive impairment and dementia: A systematic review and meta-analysis. Dement. Geriatr. Cogn. Disord..

[26] Mayne P.E., Burne T.H.J. (2019). Vitamin D in Synaptic Plasticity, Cognitive Function, and Neuropsychiatric Illness. Trends Neurosci..

[27] Holick M.F. (2004). Sunlight and vitamin D for bone health and prevention of autoimmune diseases, cancers, and cardiovascular disease. Am. J. Clin. Nutr..

[28] Holick M.F. (2007). Vitamin D deficiency. N. Engl. J. Med..

[29] Al-Daghri N.M., Hussain S.D., Ansari M.G.A., Khattak M.N.K., Aljohani N., Al-Saleh Y., Al-Harbi M.Y., Sabico S., Alokail M.S. (2021). Decreasing prevalence of vitamin D deficiency in the central region of Saudi Arabia (2008–2017). J. Steroid Biochem. Mol. Biol..

[30] Chakhtoura M., Rahme M., Chamoun N., Fuleihan G.E.H. (2018). Vitamin D in the Middle East and North Africa. Bone Rep..

[31] Gáll Z., Székely O. (2021). Role of Vitamin D in Cognitive Dysfunction: New Molecular Concepts and Discrepancies between Animal and Human Findings. Nutrients.

[32] Melough M.M., Murphy L.E., Graff J.C., Derefinko K.J., LeWinn K.Z., Bush N.R., Enquobahrie D.A., Loftus C.T., Kocak M., Sathyanarayana S. (2021). Maternal Plasma 25-Hydroxyvitamin D during Gestation Is Positively Associated with Neurocognitive Development in Offspring at Age 4–6 Years. J. Nutr..

[33] Walton C., Isaac A., Kerr M. (2019). Prevalence of vitamin D deficiency in people with learning disability: A systematic review. Br. J. Learn. Disabil..

[34] McKenzie W. Multiple Intelligences Survey. http://surfaquarium.com/MI/MIinvent.htm.

[35] Hajhashemi K., Wong B.E. (2010). A Validation Study of the Persian Version of Mckenzie’s (1999) Multiple Intelligences Inventory to Measure MI Profiles of Pre-University Students. Pertanika J. Soc. Sci. Humanit..

[36] Furnham A., Akande A. (2004). African parents’ estimates of their own and their children’s multiple intelligences. Curr. Psychol..

[37] Furnham A., Chamorro-Premuzic T. (2005). Estimating one’s own and one’s relatives’ multiple intelligence: A study from Argentina. Span J. Psychol..

[38] Furnham A., Hosoe T., Tang T.L.-P. (2002). Male hubris and female humility? A cross-cultural study of ratings of self, parental, and sibling multiple intelligence in America, Britain, and Japan. Intelligence.

[39] Barnard L., Olivarez A. (2007). Self-estimates of multiple, g factor, and school-valued intelligences. N. Am. J. Psychol..

[40] Llor L., Fernando M., Ferrandiz C., Hernandez D., Sainz M., Prietro M.D., Fernandez M.C. (2012). Inteligencias múltiples y alta habilidad [Multiple intelligences and high skills]. Aula Abierta.

[41] Beceren B.Ö. (2010). Determining multiple intelligences pre-school children (4–6 age) in learning process. Procedia Soc. Behav. Sci..

[42] AlBuhairan F.S., Tamim H., Al Dubayee M., AlDhukair S., Al Shehri S., Tamimi W., El Bcheraoui C., Magzoub M.E., de Vries N., Al Alwan I. (2015). Time for an Adolescent Health Surveillance System in Saudi Arabia: Findings From “Jeeluna”. J. Adolesc. Health.

[43] Fouda M.A., Turkestani I.Z., Almusharraf S., Al-Ajlan A., Angkaya-Bagayawa F.F., Sabico S., Mohammed A.G., Hassanato R., Al-Serehi A., Alshingetti N.M. (2017). Extremely High Prevalence of Maternal and Neonatal Vitamin D Deficiency in the Arab Population. Neonatology.

[44] Al-Othman A., Al-Musharaf S., Al-Daghri N.M., Krishnaswamy S., Yusuf D.S., Alkharfy K.M., Al-Saleh Y., Al-Attas O.S., Alokail M.S., Moharram O. (2012). Effect of physical activity and sun exposure on vitamin D status of Saudi children and adolescents. BMC Pediatrics.

[45] Wortsman J., Matsuoka L.Y., Chen T.C., Lu Z., Holick M.F. (2000). Decreased bioavailability of vitamin D in obesity. Am. J. Clin. Nutr..

[46] Cui X., Gooch H., Groves N.J., Sah P., Burne T.H., Eyles D.W., McGrath J.J. (2015). Vitamin D and the brain: Key questions for future research. J. Steroid Biochem. Mol. Biol..

[47] Al-Agha A.E., Alsharief A.A., Ahmed M.S., Nassir A.Y. (2016). The Effect of Socioeconomic Status on Vitamin D Level in Children’s and Adolescents Living at Jeddah, Saudi Arabia. Evid. Based Med. Pract..

[48] Takeuchi H., Taki Y., Asano K., Asano M., Sassa Y., Yokota S., Kotozaki Y., Nouchi R., Kawashima R. (2021). Childhood socioeconomic status is associated with psychometric intelligence and microstructural brain development. Commun. Biol..

[49] Lenhart C.M., Hanlon A., Kang Y., Daly B.P., Brown M.D., Patterson P. (2012). Gender Disparity in Structured Physical Activity and Overall Activity Level in Adolescence: Evaluation of Youth Risk Behavior Surveillance Data. Int. Sch. Res. Not..

[50] Holick M.F. (2015). Vitamin D and brain health: The need for vitamin D supplementation and sensible sun exposure. J. Intern. Med..

[51] Yates N.J., Tesic D., Feindel K.W., Smith J.T., Clarke M.W., Wale C., Crew R.C., Wharfe M.D., Whitehouse A.J.O., Wyrwoll C.S. (2018). Vitamin D is crucial for maternal care and offspring social behaviour in rats. J. Endocrinol..

[52] Freedman R., Hunter S.K., Hoffman M.C. (2018). Prenatal Primary Prevention of Mental Illness by Micronutrient Supplements in Pregnancy. Am. J. Psychiatry.

